# Correction to Developmental pharmacokinetics of indomethacin in preterm neonates: Severely decreased drug clearance in the first week of life

**DOI:** 10.1002/psp4.13289

**Published:** 2024-12-02

Krzyzanski W, Stockard B, Gaedigk A, et al. Developmental pharmacokinetics of indomethacin in preterm neonates: severely decreased drug clearance in the first week of life. *CPT Pharmacometrics Syst Pharmacol*. 2023;12:110–121. doi:10.1002/psp4.12881

In the published version of the above article, the equation reported to convert dried blood spot (DBS) indomethacin concentrations to plasma concentrations is incorrect. Rather than “plasma[IND] = DBS[IND]/(1 – hematocrit) * 1.608,” the equation should be “C(plasma) = 1.837(C(DBS)/(1 – Hct/100)) – 236.6.” There is also an inaccurate statement in the Bioanalytical methods section: “A correction factor (1.608, mean of the ratio of plasma:DBS concentrations) was used to calculate the theoretical plasma concentrations from the hematocrit‐corrected DBS concentration,” which does not align with the data analysis that was performed.

This author error in reporting does not affect the results or conclusions of the paper as the correct equation (more accurate and appropriate) was used to convert DBS to plasma concentrations for data analysis, and the wrong equation was reported in the manuscript.

The following are additional changes in the original text of the paper that reflect the accurate methods, with changes from the original methods highlighted in bold text.

(1) “To evaluate the agreement between the DBS and plasma concentrations, **32** paired DBS and plasma samples were compared.”
(2) “The theoretical plasma concentrations from DBS compared with the paired plasma data showed good agreement with a Pearson correlation coefficient of **0.98**. The theoretical plasma concentrations were compared with the direct plasma analysis via a Bland–Altman plot. The bias calculated from the Bland–Altman analysis was **−8.4%**, which was not statistically significant (95% confidence intervals [CIs] were **−76 to +59%**). The 95% CIs reflect the significant variability in using DBS data to predict plasma data; however, the Pearson coefficient indicates good accuracy.”

We apologize for this error.

